# Convergent evolution of *BRCA*2 reversion mutations under therapeutic pressure by PARP inhibition and platinum chemotherapy

**DOI:** 10.1038/s41698-024-00526-9

**Published:** 2024-02-14

**Authors:** Charlotte S. Walmsley, Philip Jonsson, Michael L. Cheng, Sean McBride, Christopher Kaeser, Herbert Alberto Vargas, Vincent Laudone, Barry S. Taylor, Rajya Kappagantula, Priscilla Baez, Allison L. Richards, Anne Marie Noronha, Dilmi Perera, Michael Berger, David B. Solit, Christine A. Iacobuzio-Donahue, Howard I. Scher, Mark T. A. Donoghue, Wassim Abida, Alison M. Schram

**Affiliations:** 1 Beth Israel Deaconess Medical Center, Boston, MA USA; 2 Harvard Medical School, Boston, MA USA; 3 Memorial Sloan Kettering Cancer Center, New York City, NY USA; 4 Loxo Oncology at Lilly, Stamford, CT USA

**Keywords:** Tumour heterogeneity, Cancer therapeutic resistance

## Abstract

Reversion mutations that restore wild-type function of the *BRCA* gene have been described as a key mechanism of resistance to Poly(ADP-ribose) polymerase (PARP) inhibitor therapy in BRCA-associated cancers. Here, we report a case of a patient with metastatic castration-resistant prostate cancer (mCRPC) with a germline *BRCA2* mutation who developed acquired resistance to PARP inhibition. Extensive genomic interrogation of cell-free DNA (cfDNA) and tissue at baseline, post-progression, and postmortem revealed ten unique *BRCA2* reversion mutations across ten sites. While several of the reversion mutations were private to a specific site, nine out of ten tumors contained at least one mutation, suggesting a powerful clonal selection for reversion mutations in the presence of therapeutic pressure by PARP inhibition. Variable cfDNA shed was seen across tumor sites, emphasizing a potential shortcoming of cfDNA monitoring for PARPi resistance. This report provides a genomic portrait of the temporal and spatial heterogeneity of prostate cancer under the selective pressure of a PARP inhibition and exposes limitations in the current strategies for detection of reversion mutations.

## Introduction

The development of targeted therapies exploiting synthetic lethality in patients with DNA damage response (DDR) defects represents an important new paradigm in the treatment of advanced solid tumors^1–5^. Poly (ADP-ribose) polymerase inhibitors (PARPi) are the most clinically advanced illustration of this, with regulatory approval for patients with ovarian, breast, pancreatic, and metastatic castration-resistant prostate cancers (mCRPC) harboring deleterious germline and/or somatic mutations in *BRCA1*, *BRCA2*, and other select circumstances^6–11^. The BRCA1 and BRCA2 proteins are required for effective homologous recombination (HR), and loss of their function leads to dependence on single-stranded DNA repair mediated by PARP. Thus, PARP inhibitors have demonstrated clinical benefit in patients with select disease types harboring *BRCA1/2* alterations.

Mutations that restore wild-type function of BRCA (reversion mutations) have been described as a mechanism of resistance to PARP and platinum inhibitor therapy^12–21^. These mutations promote therapeutic resistance by restoring the open reading frame of the mutant *BRCA* gene, or by excising the deleterious mutation, thereby re-establishing the cells’ ability to perform HR^20,22^.

Next-generation sequencing (NGS) of cell-free DNA (cfDNA) is a practical strategy for the detection of reversion mutations given the complexity of obtaining biopsy samples and the potential for spatially heterogeneous disease. Although there are clear benefits to this approach, the utility of cfDNA to detect BRCA reversion and inform therapeutic decision-making is still not well understood. Recent reports suggest that cfDNA monitoring may be helpful in the early detection of BRCA reversion mutations and could serve as a superior means of BRCA reversion detection compared to single-site tissue biopsy^12,23–28^. Here, we report the case of a patient with germline *BRCA2*-mutated prostate cancer treated with a PARPi. This report provides a genomic portrait of the temporal and spatial heterogeneity of mCRPC under the selective pressure of a PARPi and platinum chemotherapy, offering insight into the complexity of therapeutic resistance and exposing limitations in current strategies for the detection of reversion mutations.

## Results

### Case Presentation

A 56-year-old man with a germline *BRCA2* mutation (S1982Rfs*22, exon 11) was found to have a prostate-specific antigen (PSA) of 32 ng/ml and subsequently diagnosed with Gleason 9 (5 + 4) prostate adenocarcinoma metastatic to the bone and lymph nodes^29^. He was treated with androgen deprivation therapy (ADT, leuprolide) and abiraterone acetate with prednisone for eight months, followed by radical prostatectomy with retroperitoneal lymph node dissection which detected cancer in 3 of 9 lymph nodes (American Joint Committee on Cancer stage pT3bN1, Gleason 8 (4 + 4)). After a period of undetectable PSA, he was found to have a biochemical relapse. He was treated briefly with ADT followed by the nonsteroidal antiandrogen enzalutamide for twelve months with clinical benefit until a F-fluorodeoxyglucose-positron emission tomography (FDG-PET) scan revealed a new pelvic soft tissue mass and growth in osseous rib and spinal lesions, prompting biopsy and treatment discontinuation.

**Fig. 1:**
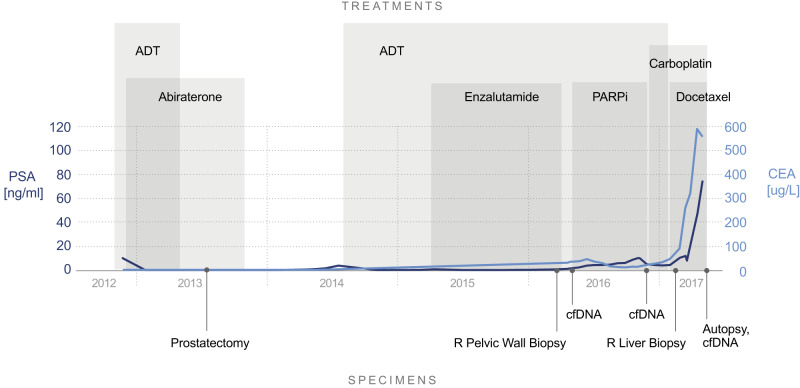
Clinical timeline including tumor markers, research sample collections, and treatments. Solid circles represent specimen collection timepoints. Light blue line indicates CEA levels. Dark blue line indicates PSA levels. PSA prostate-specific antigen, CEA carcinoembryonic antigen, cfDNA cell-free DNA, ADT androgen deprivation therapy, PARPi Poly(ADP-ribose) polymerase inhibitor combination.

Following written informed consent, the patient was initiated on a PARPi combination through an IRB-approved protocol. He experienced tumor shrinkage with prolonged stable disease by RECIST v1.1. PSA slowly rose throughout treatment from 2 ng/ml to 10 ng/ml, while carcinoembryonic antigen (CEA) declined from 36 ng/ml nadiring at 11 ng/ml four months into therapy. Six months after treatment initiation the patient developed clinical disease progression. cfDNA sequencing using MSK-ACCESS, an ultra-high coverage NGS panel that includes exons from 129 cancer-associated genes, including all exons of *BRCA2*, did not detect any resistance mechanism compared to baseline cfDNA^30^. The patient received carboplatin every three weeks for two months with an initial on-treatment scan showing disease stability followed by rapid progression in the liver, pelvis, lymph nodes, and bone. While PSA decreased on carboplatin, CEA levels rose (Fig. 1). Prostate-specific membrane antigen (PSMA) PET scan demonstrated high PSMA expression in the bone, lymph nodes, and pelvis without any expression in the liver metastases. A biopsy of the liver demonstrated poorly differentiated adenocarcinoma, PSA-negative but consistent with a prostate primary. NGS using MSK-IMPACT, a hybrid capture-based NGS assay that sequences all exons of 468 cancer-associated genes, revealed an acquired *BRCA2* intragenic deletion of exon 11 predicted to excise the germline mutation and restore the *BRCA2* reading frame to recreate a functional protein^31^.

**Fig. 2:**
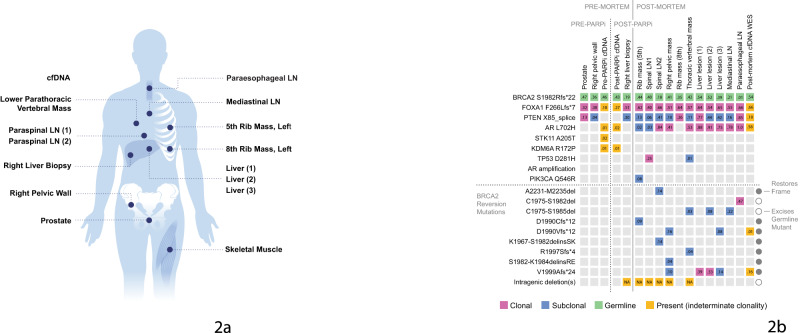
Integrated analysis of sample collection and genomic alterations at multiple timepoints: schematic and genomic landscape from pre to post-PARPi and autopsy. **a** Schematic illustrating the sites of blood and tissue sample collections from multiple timepoints including pre and post-mortem. **b** Genetic alterations and variant allele frequencies across sample sites, including cfDNA and tissue samples collected pre- and post-PARPi and at time of autopsy. Tumors were subjected to extensive genomic interrogation using MSK-IMPACT. cfDNA was analyzed using either MSK-ACCESS (baseline and progression cfDNA), or WES (autopsy cfDNA). Note, all disease-relevant and oncogenic mutations detected in the MSK-ACCESS, MSK-IMPACT, and WES panels are included above with the exception of mutations seen in cfDNA with variant allele frequencies <1%, as these were considered possible clonal hematopoiesis (pre-PARPi cfDNA: PIK3R1 D464-Y467del, BRCA1 T1485S, post-PARPi cfDNA: TP53 C176Y, TP53 X126_splice).

The patient received carboplatin and docetaxel for two months with rapid disease progression resulting in death. The family graciously consented to a research autopsy and future publication. Tissue and cfDNA samples were collected, including 19 samples from lesions in the ribs, pelvis, vertebra, liver, lymph nodes, and skeletal muscle as a matched normal, eleven of which were sent for genomic analysis (Fig. 2a).

To further elucidate the mechanisms of resistance to PARP combination therapy, we completed extensive genomic interrogation of cfDNA and tissue pre- and post-PARPi and post-mortem. cfDNA collected at baseline and progression on PARPi therapy was sequenced using MSK-ACCESS, with an average depth of >20,000X^30^. A cfDNA sample collected at the time of autopsy was analyzed by whole exome sequencing (WES) to an average depth of 290X. The calculated tumor fraction in this sample was 0.57^32^. WES was performed on multiple metastatic sites using previously described methods^32^.

Genomic sequencing of 11 sites of metastatic disease demonstrated extensive intratumoral heterogeneity, with ten unique *BRCA2* reversion mutations across ten sites (Fig. 2a). The 10 reversion mutations identified on autopsy are predicted to re-establish *BRCA2* function by restoring the open reading frame (7 mutations) or excising the germline mutation (3 mutations). None of the reversion mutations were detected in the post-PARPi cfDNA sequenced with MSK-ACCESS, and only two of ten reversion mutations were detected in the post-mortem cfDNA WES sample. The intragenic deletion identified pre-mortem was only seen in five of 10 specimens. Six reversion mutations were unique to a single metastatic site. No one mutation was present across all specimens.

## Discussion

PARP inhibitors are standard of care for patients with *BRCA1/2*-mutated ovarian, breast, pancreatic, and mCRPC based on several well-conducted clinical trials demonstrating efficacy in these populations^10,33–40^. Unfortunately, primary and acquired resistance to treatment ultimately limits clinical benefit. Reversion mutations have been described in a minority of patients with acquired PARPi resistance. Here we report the results of extensive genomic interrogation of a *BRCA2*-mutant prostate cancer patient with acquired resistance to PARPi combination therapy mediated by multiple *BRCA2* reversion mutations.

This case is the most extensive analysis of autopsy and longitudinal clinical specimens for PARPi-acquired resistance in *BRCA*-mutant mCRPC to date, and a remarkable demonstration of spatial heterogeneity and convergent evolution. Genomic analysis revealed ten unique *BRCA2* reversion mutations across ten sites. While several of the reversion mutations were private to a specific site, nine out of ten tumors contained at least one mutation, suggesting a powerful clonal selection for reversion mutations in the presence of therapeutic pressure by PARP inhibition.

The breadth of analysis in this case including both tumor and liquid biopsies from multiple timepoints sheds new light on the limitations of single-site biopsies or cfDNA as a comprehensive method to detect reversion mutations. The pre-mortem liver biopsy did not capture the full spectrum of spatially heterogeneous mutations present in this patient. Moreover, cfDNA collected post-PARPi and post-mortem did not reveal the majority of *BRCA2* reversion mutations, suggesting differential shed from distinct anatomic sites. The germline and clonal somatic mutations present at the time of autopsy (*BRCA2* germline, *FOXA1, PTEN* and *AR* somatic) were detected in the cfDNA collected post-mortem, but only 13% (2/15) of spatially heterogeneous mutations were detected. Recent reports suggest that cfDNA provides a more comprehensive summary of spatial and temporal heterogeneity when compared to single tissue biopsy^12,23–25,27,28^, however, cfDNA can be limited in identifying non-truncal mutations, especially if the mutations are confined to a single lesion^25,26,41^.

False negatives in plasma-based assays can be caused by several factors, most commonly low circulating tumor fraction, and sequencing sensitivity^42,43^. Short cfDNA half-life and varying tumor shed from distinct sites can also play a role^42,43^. The cfDNA sample collected immediately post-PARPi in this case was of good quality, with target coverage of 20,000X, suggesting these results can be extrapolated to commercial cfDNA assays and therefore routine clinical care. Furthermore, the clonal FOXA1 mutation was observed with a VAF of 26.6%, confirming the presence of a high proportion of circulating tumor DNA. Notably, the cfDNA sample collected post-mortem was sequenced using WES and therefore had lower coverage of *BRCA2* than the post-PARPi sample sequenced by MSK-ACCESS. Lower sensitivity in the WES cfDNA sample may have contributed to the lack of reversion mutations detected in the post-mortem sample. This reflects the common tradeoff between the breadth and depth of coverage in an assay and reinforces the importance of choosing a methodology consistent with the clinical question. For example, if identification of subclonal on-target resistance is the goal, a more limited but sensitive assay is preferable; however, WES may provide a more complete genomic picture. Ultimately, the variability of cfDNA shed across tumor sites in this case and increased assay sensitivity required to identify spatially heterogeneous mutations emphasizes a potential shortcoming of cfDNA monitoring for PARPi resistance. This challenges prior reports suggesting that cfDNA may be a reliable method for reversion mutation monitoring in this clinical setting^24,26^.

The frequency of *BRCA* reversion mutations is still not well understood. A recent meta-analysis estimates the rate of *BRCA2* reversion mutations post-PARP inhibitor or platinum therapy as 30.7% across all cancer types^32^. However, many studies use cfDNA as the detection method for reversion mutations, which may underestimate the true occurrence due to limitations of cfDNA in detecting non-truncal mutations, deletions, and more complex genomic alterations^25,43,44^.

Identifying *BRCA* reversion mutations is clinically important for several reasons. In addition to predicting for resistance to PARP inhibitors, the presence or absence of a reversion mutation may help inform the choice of chemotherapy. The patient in this study received minimal clinical benefit from platinum-based chemotherapy after acquired resistance to PARP inhibition, which supports the previously described cross-resistance between PARP inhibitors and platinum-based therapies in the presence of BRCA reversion mutations^45^. Although it is possible that one or more of the reversion mutations arose during platinum treatment as has been previously described^46^, the rapid progression on carboplatin suggests that the reversion mutations likely pre-existed from the selective pressure of PARPi, despite cfDNA post-PARPi failing to detect any reversion mutations. Further research is needed on the frequency of *BRCA* reversion mutations as a mechanism of platinum resistance in the setting of mCRPC. Repeat biopsy and/or cfDNA acquisition at the time of acquired resistance to PARP inhibitors may therefore inform future therapy, although negative cfDNA results should be interpreted with caution. While the presence of a reversion mutation is useful for clinical decision-making, the absence of such a mutation does not exclude the possibility that one exists and repeat cfDNA testing, tissue testing, and clinical cues may be helpful in this context.

In conclusion, these data indicate that PARP inhibitors exert a powerful selective pressure for *BRCA* reversion mutations in *BRCA*-mutant tumors, as evidenced by extensive convergent evolution in a patient with *BRCA2*-mutant prostate cancer. Identification of these resistance mechanisms may help guide therapeutic decision-making. Our case also suggests that cfDNA may fail to appropriately reveal *BRCA* reversion mutations, a phenomenon that has likely been underestimated in the current literature.

## Methods

### Participant

The patient was treated with combination therapy including a PARP inhibitor through a prospective IRB-approved research protocol. The patient provided written informed consent for genomic sequencing of germline tissue, tumor and cfDNA, and review of medical records for detailed demographic, pathologic, and clinical data as part of an institutional IRB-approved research protocol (MSKCC; NCT01775072). The family consented to a research autopsy and future publication. Research protocols for tumor collection and analysis were approved by the ethical committees of MSKCC. The study was conducted in accordance with all relevant ethical regulation including the Declaration of Helsinki.

### Sample acquisition

Tumor samples were obtained by core biopsy and rapid autopsy. Core biopsy samples were collected by an interventional radiologist. Rapid autopsy samples were collected by a team consisting of a pathologist, medical oncologist, and pathology assistant as previously described^47^. Briefly, harvested samples were collected from metastatic sites and either formalin-fixed for paraffin embedding and routine histologic examination or flash-frozen in liquid nitrogen and stored at −80 °C. cfDNA was extracted from plasma (MagMAX cfDNA isolation kit) and buffy coat (Chemagen magnetic bead technology)^30^.

### Library preparation and sequencing

Sample preparation, processing, read alignment and variant calling of premortem cfDNA samples^30^ and tumor samples^31^ were performed as previously described. Briefly, cfDNA was extracted from plasma, UMI-tagged libraries constructed using xGen Duplex Seq Adapters with dual index barcodes (IDT, Integrated DNA Technologies) before pooling and capture with custom xGen Lockdown probes (IDT). Samples using MSK-ACCESS were sequenced at an average depth of >20,000X^30^. Captured DNA fragments were sequenced on an Illumina sequencer (HiSeq 2500 or NovaSeq 6000) as paired-end reads. Samples using whole exome sequencing (WES) were sequenced to an average depth of 290X. Analysis of tissue and post-mortem cfDNA WES samples was performed as previously described^32^. In brief, target capture was performed using SureSelect Human All Exon V6 (Agilent Technologies) and sequenced on HiSeq 2500 sequencer (Illumina).

### Computational analyses

All sequencing data were processed as previously described^30–32^. In brief, read processing and alignment were performed using GATK best practices with ABRA^48^ for indel realignment. UMIs for cfDNA samples were handled using Marianas (https://github.com/mskcc/Marianas) to produce error-suppressed consensus reads. MuTect, VarDict, Strelka and HaplotypeCaller were used for somatic variant calling, and VEP and OncoKB^49^ for subsequent variant annotation and prioritization of variants with likely oncogenic effect. Inferred tumor purity and copy number states from Facets were used to generate clonality estimates from observed variant allele fractions, as described previously^32,50^. Mutations in Fig. 2B were manually reviewed in all samples using IGV.

### Reporting summary

Further information on research design is available in the Nature Research Reporting Summary linked to this article.

### Supplementary information

REPORTING SUMMARY

## Data Availability

All whole-exome sequencing data as well as germline calls were deposited to the National Center for Biotechnology Information Genotype and Phenotype Database (NCBI dbGaP). Accession number phs003423.v1.p1. Due to the nature of the informed consent document, genomic summary results from this study are only made available through controlled access.
