# Supplementary material for: Convergent evolution of BRCA2 reversion mutations under therapeutic pressure by PARP inhibition and platinum chemotherapy
Source: NPJ Precis Oncol. 2024 Feb 14;8:34. doi: 10.1038/s41698-024-00526-9 (PMC10866935; doi:10.1038/s41698-024-00526-9)
Supplement: Supplementary file 1 — REPORTING SUMMARY [file 41698_2024_526_MOESM1_ESM.pdf]

nature portfolio

Corresponding author(s): Alison Schram, schrama@mskcc.org

Last updated by author(s): 11/29/2023

Reporting Summary

Nature Portfolio wishes to improve the reproducibility of the work that we publish. This form provides structure for consistency and transparency in reporting. For further information on Nature Portfolio policies, see our Editorial Policies and the Editorial Policy Checklist.

Statistics

For all statistical analyses, confirm that the following items are present in the figure legend, table legend, main text, or Methods section.

n/a	Confirmed	
☒	☐	The exact sample size (n) for each experimental group/condition, given as a discrete number and unit of measurement
☒	☐	A statement on whether measurements were taken from distinct samples or whether the same sample was measured repeatedly
☒	☐	The statistical test(s) used AND whether they are one- or two-sided<br>*Only common tests should be described solely by name; describe more complex techniques in the Methods section.*
☒	☐	A description of all covariates tested
☒	☐	A description of any assumptions or corrections, such as tests of normality and adjustment for multiple comparisons
☒	☐	A full description of the statistical parameters including central tendency (e.g. means) or other basic estimates (e.g. regression coefficient) AND variation (e.g. standard deviation) or associated estimates of uncertainty (e.g. confidence intervals)
☒	☐	For null hypothesis testing, the test statistic (e.g. F, t, r) with confidence intervals, effect sizes, degrees of freedom and P value noted<br>*Give P values as exact values whenever suitable.*
☒	☐	For Bayesian analysis, information on the choice of priors and Markov chain Monte Carlo settings
☒	☐	For hierarchical and complex designs, identification of the appropriate level for tests and full reporting of outcomes
☒	☐	Estimates of effect sizes (e.g. Cohen's d, Pearson's r), indicating how they were calculated

Our web collection on statistics for biologists contains articles on many of the points above.

Software and code

Policy information about availability of computer code

Data collection	N/A
Data analysis	N/A

For manuscripts utilizing custom algorithms or software that are central to the research but not yet described in published literature, software must be made available to editors and reviewers. We strongly encourage code deposition in a community repository (e.g. GitHub). See the Nature Portfolio guidelines for submitting code & software for further information.

Data

Policy information about availability of data

All manuscripts must include a data availability statement. This statement should provide the following information, where applicable:

- Accession codes, unique identifiers, or web links for publicly available datasets
- A description of any restrictions on data availability
- For clinical datasets or third party data, please ensure that the statement adheres to our policy

All whole-exome sequencing data as well as germline calls were deposited to the National Center for Biotechnology Information Genotype and Phenotype Database (NCBI dbGaP). Accession number phs003423.v1.p1. Due to the nature of the informed consent document, genomic summary results from this study are only made available through controlled access.

Human research participants

Policy information about studies involving human research participants and Sex and Gender in Research.

Reporting on sex and gender	Our case report includes one human research participant of male sex. His gender is not reported in this study. The patient provided written informed consent for genomic sequencing of germline tissue, tumor and cfDNA, and review of medical records for detailed demographic, pathologic, and clinical data as part of an institutional IRB-approved research protocol (MSKCC; NCT01775072).
Population characteristics	Our case report includes one human research participant: a 56-year-old man with a germline BRCA2 mutation (S1982Rfs*22, exon 11) diagnosed with Gleason 9 (5+4) prostate adenocarcinoma metastatic to the bone and lymph nodes. He was treated with androgen deprivation therapy (ADT, leuprolide) and abiraterone acetate with prednisone for eight months, followed by radical prostatectomy Subsequently, he was treated briefly with ADT followed by the nonsteroidal antiandrogen enzalutamide for twelve months. Following written informed consent, the patient was initiated on a PARPi combination through an IRB-approved protocol followed by carboplatin and docetaxel.
Recruitment	The patient was recruited to the study
Ethics oversight	IRB approved protocol at MSKCC; NCT01775072

Note that full information on the approval of the study protocol must also be provided in the manuscript.

Field-specific reporting

Please select the one below that is the best fit for your research. If you are not sure, read the appropriate sections before making your selection.

☒ Life sciences ☐ Behavioural & social sciences ☐ Ecological, evolutionary & environmental sciences

For a reference copy of the document with all sections, see nature.com/documents/nr-reporting-summary-flat.pdf

Life sciences study design

All studies must disclose on these points even when the disclosure is negative.

Sample size	N/A
Data exclusions	N/A
Replication	N/A
Randomization	N/A
Blinding	N/A

Reporting for specific materials, systems and methods

We require information from authors about some types of materials, experimental systems and methods used in many studies. Here, indicate whether each material, system or method listed is relevant to your study. If you are not sure if a list item applies to your research, read the appropriate section before selecting a response.

Materials & experimental systems

n/a	Involved in the study	
☒	☐	Antibodies
☒	☐	Eukaryotic cell lines
☒	☐	Palaeontology and archaeology
☒	☐	Animals and other organisms
☒	☐	Clinical data
☒	☐	Dual use research of concern

Methods

n/a	Involved in the study	
☒	☐	ChIP-seq
☒	☐	Flow cytometry
☒	☐	MRI-based neuroimaging
